# Performance Evaluation of the Newly Developed In Vitro Rapid Diagnostic Test for Detecting OXA-48-Like, KPC-, NDM-, VIM- and IMP-Type Carbapenemases: The RESIST-5 O.K.N.V.I. Multiplex Lateral Flow Assay

**DOI:** 10.3390/antibiotics10040460

**Published:** 2021-04-19

**Authors:** Junsung Hong, Dayoung Kang, Dokyun Kim

**Affiliations:** 1 Department of Laboratory Medicine, College of Medicine, Yonsei University, Seoul 06273, Korea; jumphong2@nate.com (J.H.); ideakang1@naver.com (D.K.); 2 Research Institute of Bacterial Resistance, College of Medicine, Yonsei University, Seoul 06273, Korea

**Keywords:** carbapenemase, evaluation, carbapenemase-producing organisms, IMP, VIM, NDM, KPC, OXA-48-like, RESIST-5 O.K.N.V.I. assay

## Abstract

The objective of this study was to evaluate the performance of the RESIST-5 O.K.N.V.I. assay for identifying these five common domestic carbapenemases among a large number of clinical isolates in South Korea. A total of 268 non-duplicated clinical isolates of gram-negative bacilli were included in this study as follows: 258 carbapenemase-producing (CP) strains (OXA-48-like, KPC, NDM, VIM, IMP, GES, OXA-23 and two or more carbapenemase producers) and 10 non-CP carbapenem-resistant *Enterobacterales* (non-CP CREs). Overall sensitivity and specificity were 98.4% and 100%, respectively. In addition, all non-targeted carbapenemase producers including GES and OXA-23 producers and non-CP CREs were correctly identified as negative results. There were only four discrepant cases in which three VIM carbapenemase producers and one NDM carbapenemase producer were not detected. The RESIST-5 O.K.N.V.I. assay as an in vitro diagnostic test for detecting five common carbapenemases provided rapid and accurate results in a short time, indicating that this method could provide an innovative solution for early detection, resulting in appropriate antimicrobial treatment in the clinical field.

## 1. Introduction

The dissemination of carbapenem-resistant organisms is a serious global threat to human public health with few treatment options for infected patients due to their co-resistance to other β-lactam antimicrobials [1,2]. Notably, the genes encoding carbapenemases, which were reported to be one of the major mechanism for carbapenem resistance, are mostly located on mobile genetic elements such as transposons, plasmids and genomic islands. Thus, horizontal transfer of these genes frequently occurs among bacterial species [3]. Therefore, carbapenemase-producing (CP) organisms, including *Enterobacterales* and glucose-non-fermenting bacilli (GNFB), have become widespread in several countries, including South Korea [4,5]. The rapid and accurate detection and identification of carbapenemases to prevent further dissemination and to address adequate antimicrobial treatment of infected patients in the clinical field remain a challenge [6].

Among the diverse types of carbapenemases, the five most prevalent enzymes in *Enterobacterales* and GNFB isolates in South Korea include KPC variants of Ambler class A, three metallo-β-lactamases (MBLs) of Ambler class B (NDM, VIM and IMP-variants) and OXA-48-like-variants of Ambler class D [7]. For identifying and characterizing the variable types of carbapenemases, several diagnostic tools, such as culture-based methods using resistant phenotypes and molecular biology-based methods using gene amplification, have been widely used in clinical microbiology laboratories [8,9,10]. However, culture-based phenotypic methods are labor intensive and time consuming and the molecular method needs expensive equipment and high expertise. Recently, multiplex immunochromatographic lateral flow assays for detecting and characterizing carbapenemases were developed [11,12] and the RESIST-5 O.K.N.V.I. assay (CORIS BioConcept, Gembloux, Belgium) with membrane technology of colloidal gold nanoparticles was introduced to identify five targeted carbapenemase genes in a single test without specialized equipment within 15 min.

In this study, we evaluated the performance of the RESIST-5 O.K.N.V.I. assay for detecting the five common carbapenemases (OXA-48-like, KPC, NDM, VIM and IMP) compared to conventional PCR and sequencing analysis among a large number of clinical isolates in South Korea.

## 2. Materials and Methods

### 2.1. Study Design

A total of 268 non-duplicated clinical isolates of gram-negative bacilli, composed of 258 CP organisms (KPC producers [*n* = 40], GES producers [*n* = 15], NDM producers [*n* = 41], VIM producers [*n* = 42], IMP producers [*n* = 45], OXA-48-like producers [*n* = 40], OXA-23 producers [*n* = 15] and two or more carbapenemase producers [*n* = 20]) and 10 non-CP, but carbapenem-resistant *Enterobacterales* (non-CP CREs), were used to evaluate the performance of the RESIST-5 O.K.N.V.I. assay kit. The detailed information of tested isolates including the origin of the isolates is presented in Supplementary Table S1.

### 2.2. Bacterial Identification

Bacterial identification was performed with MALDI MS using a Bruker MALDI MS instrument (Bruker, Billerica, MA, USA) and all the isolates were characterized with conventional PCR and sequence analysis as reference molecular methods.

### 2.3. Conventional PCR Method

Bacterial DNA was extracted by the thermal lysis method from colonies grown on MacConkey agar. The PCR was performed under the following amplification conditions: 94 °C for 5 min, followed by 30 cycles at 94 °C for 30 s, then 58 °C (*bla*_KPC_) or 60 °C (*bla*_IMP_, *bla*_NDM_, *bla*_VIM_ and *bla*_OXA-48-like_) for 30 s and 72 °C for 30 s, followed by a final extension at 72 °C for 5 min using C1000^TM^ Thermal Cycler (Bio-Rad, Hercules, CA, USA) (Table 1) [3]. The primers used in this study are summarized in Table S2.

### 2.4. RESIST-5 O.K.N.V.I. Assay

**Figure 1:**
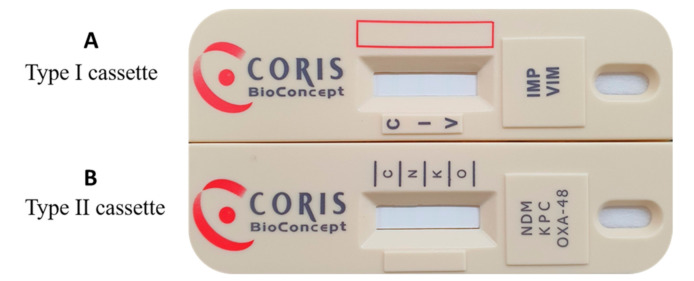
Two lateral-flow cassettes. Each cassette contains one sensitized strip. (**A**) The type I cassette contains a nitrocellulose membrane sensitized with monoclonal antibody directed against IMP carbapenemase (“I” line) and VIM carbapenemase (“V” line) and a control capture reagent (“C” line). (**B**) The type II cassette contains a nitrocellulose membrane sensitized with monoclonal antibody directed against NDM carbapenemase (“N” line), KPC carbapenemase (“K” line) and OXA-48-like carbapenemase (“O” line) and a control capture reagent (“C” line).

The RESIST-5 O.K.N.V.I. assay (CORIS BioConcept, Gembloux, Belgium) is a new immunochromatography test composed of two lateral-flow cassettes (one cassette for VIM and IMP and the other cassette for OXA-48-like, KPC and NDM) for identification of five targeted carbapenemases (Figure 1). These tests are based on a membrane technology with different colloidal gold nanoparticles. A nitrocellulose membrane is sensitized with each monoclonal antibody directed against OXA-48-like, KPC, NDM, VIM and IMP carbapenemases and their variants.

The procedure for the RESIST-5 O.K.N.V.I. assay is as follows: (i) Prepare one semi-rigid tube and add 12 drops of LY-A buffer (saline solution buffered to pH 7.5 containing TRIS, NaN3 [<0.1%] and a detergent), (ii) Harvest the bacteria by collecting one colony on MacConkey agar (BANDIO, Gyeonggi-do, Korea) with a disposable bacteriological loop and dip the loop in the bottom of the semi-rigid tube containing the buffer, (iii) Stir thoroughly before removing the loop, (iv) Insert the dropper tightly on the semi-rigid tube, (v) Vortex the preparation to homogenize (the entire bacterial colony must be suspended in the buffer), (vi) Invert the test tube and slowly add three drops of diluted sample into each well of the two cassettes labelled (i) NDM, KPC and OXA-48 and (ii) IMP and VIM (alternatively, add 100 µl of diluted sample with a micropipette to each cassette sample well) and (vii) Allow to react for 15 min maximum and read the result.

The results are interpreted as follows for both cassettes. Negative test results are indicated by a reddish-purple line at the Control line (C) position with no other line present. Positive test results are indicated by a visible reddish-purple test line at OXA-48-like (“O” line), KPC (“K” line), NDM (“N” line), VIM (“V” line) and/or IMP (“I” line) in addition, to a line at the “C” line. A weak line should be considered a positive result. The results of the RESIST-5 O.K.N.V.I. assay were blindly interpreted by a highly trained laboratory technician.

## 3. Results

### 3.1. Identification of Single Carbapenemase Producers

Table 2 demonstrates the comparison between the results of the RESIST-5 O.K.N.V.I. assay and the conventional method for detecting carbapenemases used in this study. All 40 OXA-48-like-producing *Klebsiella pneumoniae* isolates carrying the *bla*_OXA-232_ gene were correctly identified by the RESIST-5 O.K.N.V.I. assay compared to the conventional method. In addition, 40 KPC-type carbapenemase-producing organisms including 27 *K. pneumoniae*, eight *E. coli*, three *Enterobacter*, one *Klebsiella oxytoca* and one *Serratia marcescens* isolate were correctly identified irrespective of KPC variant (KPC-2, KPC-3 and KPC-4). All 45 IMP-1-, IMP-4-, IMP-6- and IMP-10-producing clinical isolates including 38 *Pseudomonas aeruginosa*, four *Enterobacter* and three *K. pneumoniae* isolates were correctly identified. Among the 41 NDM-producing clinical isolates including 22 *E. coli*, nine *Enterobacter*, four *K. pneumoniae*, four *Citrobacter freundii*, one *Citrobacter amalonaticus* and one *K*. *oxytoca* isolate with different NDM variants including NDM-1, NDM-5, NDM-7 and NDM-9, only one (GNSEV_NDM_38: *E. coli* carrying the *bla*_NDM-1_ gene) showed a false negative result. Finally, three of 42 VIM-producing clinical isolates including two VIM-1 producing *K. pneumoniae* isolates (GNSEV_VIM_10 and GNSEV_VIM_32) and one VIM-2 producing *Enterobacter* isolate (GNSEV_VIM_5) showed false negative results.

To evaluate the four discrepancies, minimum inhibitory concentrations (MICs) were determined by the broth microdilution method. However, the MICs of imipenem and meropenem of the GNSEV_NDM_38 strain were 4 mg/L and 8 mg/L, respectively, which was similar to that of other NDM-producing isolates in this study (imipenem MIC_50_ = 4 mg/L; meropenem MIC_50_ = 4 mg/L). Three VIM-producing clinical isolates exhibiting false-negative results showed similar MICs of carbapenems to other VIM-producing isolates.

### 3.2. Identification of Multiple Carbapenemase Producers

To evaluate the performance of the RESIST-5 O.K.N.V.I. assay in detecting multiple carbapenemase producers, 20 clinical *Enterobacterales* carrying two or more carbapenemase genes were tested in this study. The isolates carried six major carbapenemase variants including combinations of KPC-, GES-, NDM-, VIM-, IMP- and/or OXA-48-like carbapenemases (Table 1) and all isolates were correctly identified compared with the conventional methods.

### 3.3. Identification of Non-Targeted Carbapenemase Producers and Non-CP CRE Isolates

All 15 GES-producing clinical isolates including seven *P. aeruginosa*, six *Enterobacter* and two *K. pneumoniae* isolates and 15 OXA-23-producing *Acinetobacter baumannii* were correctly identified as negative results. In addition, 10 non-CP CRE isolates, which did not carry five targeted carbapenemases, also showed negative results.

The overall sensitivity, specificity, positive predictive values and negative predictive values of RESIST-5 O.K.N.V.I. for detecting the five targeted carbapenemases (OXA-48-like, KPC, NDM, VIM and IMP) in this study are presented in Table 3.

## 4. Discussion

Outbreaks of CP organisms carrying KPC-type, MBLs (NDM, VIM and IMP carbapenemase), or OXA-type carbapenemases have been reported in many countries and they are associated with healthcare- and community-acquired infections [1,2,4]. In South Korea, the most dominant type of carbapenemase is the KPC-type, followed by NDM, OXA-48-like, VIM and IMP carbapenemase in *Enterobacterales* [7]. Therefore, rapid and accurate detection of these five major resistance determinants for carbapenems in clinical isolates is important for appropriate antimicrobial treatment in infected patients and for prevention of spread of infection [13]. The gold-standard method of conventional PCR and sequencing can accurately derive the quantity and subtype of a carbapenemase gene, though determination of the type of carbapenemase is enough to choose appropriate antimicrobial treatment in infected patients.

There are several types of diagnostic kits or methods for detecting carbapenemases from bacterial hosts: (i) carbapenemase inhibition test (CIM) [14], Carba NP test (bioMérieux, Marcy-l’Étoile, France) [6] and BD Phoenix^TM^ CPO detect test (BD Diagnostic Systems, Sparks, MD, USA) [15] for phenotypic detection; (ii) Xpert-Carba-R assay test (Cepheid, Sunnyvale, CA, USA) [16] and PANA RealTyper^TM^ CRE kit test (PANAGENE, Daejeon, South Korea) [10] for molecular detection; and (iii) Carba 5 assay (NG Biotech, Guipry, France) [12] for antibody POCT multiplex immunochromatographic lateral flow assays. According to previous reports, the CIM and Carba NP showed high concordance rates (100% for *Enterobacterales* and 98.8% for non-fermenters for both methods) with conventional methods in CP organisms [17]. The BD Phoenix^TM^ CPO detection kit identified carbapenemase producers with 97.2% overall agreement rate and it classified Ambler class A (81.7%), class B (71.8%) and class D (82.0%) carbapenemase producers [15]. For the molecular detection method, the Xpert Carba-R assay showed overall 100% sensitivity for detecting five carbapenemases (OXA-48-like, KPC, NDM, VIM and IMP), [16] and the PANA RealTyper^TM^ CRE kit showed 98.9% to 100% sensitivity for detecting six carbapenemases (OXA-48-like, KPC, GES, NDM, VIM and IMP) [10]. The Carba 5 assay reached 100% sensitivity for detecting five carbapenemases (OXA-48-like, KPC, NDM, VIM and IMP) [12]. However, the tests based on phenotypic methods are often ambiguous in interpretation and time-consuming due to incubation period and molecular methods require expensive equipment and skilled personnel. Therefore, they have limitations in general applications. In these regards, the newly developed RESIST-5 O.K.N.V.I. assay based on multiplex immunochromatographic lateral flow assays has the following advantages: (i) A single, unique test for detecting five clinically relevant carbapenemases, (ii) no special equipment required, (iii) easy and clear interpretation and (iv) short time-to-result.

The RESIST-4 O.K.N.V. assay was introduced in 2019 and showed excellent performance in detecting the four carbapenemases, OXA-48-like, KPC, NDM and VIM [11]. Recently, the RESIST-5 O.K.N.V.I. assay was released for detecting five carbapenemases including the additional IMP carbapenemase. In a retrospective study performed on 164 non-duplicated CP *Enterobacterales* in the National Reference Laboratory for Multidrug-Resistant Gram-Negative Bacilli in Belgium, KPC and OXA-48-like carbapenemases were correctly detected with the RESIST-5 O.K.N.V.I. assay and the sensitivity for detection of the NDM, VIM and IMP carbapenemases was 91.2% (31/34), 90% (36/40) and 84.2% (16/19), respectively [18]. In our study, all OXA-48-like, KPC and IMP carbapenemase producers were correctly identified and each carbapenemase in all 20 multiple carbapenemase producers included in this study was correctly detected by the RESIST-5 O.K.N.V.I. assay. In addition, all non-targeted carbapenemase producers including GES- and OXA-23-producing isolates were correctly identified as negative results, which indicates no cross-reactivity between Ambler class carbapenemases. Non-CP CRE isolates exhibiting resistance to carbapenems (disk diffusion zone diameter: 16–23 mm for imipenem and 16–23 mm for meropenem) were correctly identified as negative results.

In this study, only four false negative results were identified including one NDM and three VIM producers compared to the results of conventional PCR and sequencing method. VIM carbapenemase has strong hydrolysis activity to carbapenems, but their susceptibility to carbapenems has been oddly documented as low [7,19]. Inaccurate detection of VIM carbapenemase was previously reported in a study on the BD Phoenix^TM^ CPO detect test with a correct classification rate of 58.6% [15]. The inaccurate detection of VIM carbapenemase by the RESIST-5 O.K.N.V.I. assay is probably related to low-level expression of purified recombinant protein [20].

Compared with other previous studies, one of the advantages of this study is that a large number of carbapenemase-producing isolates, including more than 40 clinical isolates of each type of carbapenemase producers with many variant genotypes, was included [11,12,17]. Another advantage is that the control group was discreetly selected and included two domestic, non-targeted, OXA-23 and GES carbapenemase-producing clinical isolates as well as non-CP CRE clinical isolates to evaluate cross-reactivity among other Ambler class A and Ambler class D carbapenemases, which are disseminated in South Korea [7,21].

## 5. Conclusions

Our results suggest that the RESIST-5 O.K.N.V.I. assay has excellent performance in detection of five targeted carbapenemase genes. In addition, the RESIST-5 O.K.N.V.I. assay is a rapid, easy and efficient tool to apply in the clinical microbiology laboratory.

## Figures and Tables

**Table 1:** The 268 clinical isolates included in this study.

Carbapenemase (*n*)	Variants (*n*)	Species (*n*)
KPC (40)	KPC-2 (20)	*K. pneumoniae* (10), *E. coli* (6), *Enterobacter* species (2), *K. oxytoca* (1), *S. marcescens* (1)
	KPC-3 (5)	*K. pneumoniae* (4), *E. coli* (1)
	KPC-4 (15)	*K. pneumoniae* (13), *E. coli* (1), *Enterobacter* species (1)
GES (15)	GES-5 (14)	*Enterobacter* species (6), *P. aeruginosa* (6), *K. pneumoniae* (2),
	GES-24 (1)	*P. aeruginosa* (1)
IMP (45)	IMP-1 (4)	*K. pneumoniae* (3), *Enterobacter* species (1)
	IMP-4 (3)	*Enterobacter* species (3)
	IMP-6 (37)	*P. aeruginosa* (37)
	IMP-10 (1)	*P. aeruginosa* (1)
NDM (41)	NDM-1 (30)	*Enterobacter* species (9), *E. coli* (12), *K. pneumoniae* (4), *C. freundii* (3), *K. oxytoca* (1), *C. amalonaticus* (1)
	NDM-5 (8)	*E. coli* (7), *C. freundii* (1)
	NDM-7 (2)	*E. coli* (2)
	NDM-9 (1)	*E. coli* (1)
VIM (42)	VIM-1 (8)	*K. pneumoniae* (4), *Enterobacter* species (3), *C. freundii* (1)
	VIM-2 (34)	*Enterobacter* species (23), *K. pneumoniae* (3), *P. aeruginosa* (3), *S. marcescens* (2), *C. freundii* (1), *E. coli* (1), *K. oxytoca* (1),
OXA-48-like (40)	OXA-232 (40)	*K. pneumoniae* (40)
Co-producers (20)	KPC-2 and NDM-1 (6)	*K. variicola* (2), *C. freundii* (2), *K. oxytoca* (1), *R. ornithinolytica* (1),
	KPC-3 and NDM-1 (3)	*Raoultella planticola* (3)
	KPC-4 and NDM-1 (1)	*K. pneumoniae* (1)
	KPC-4 and NDM-5 (1)	*K. pneumoniae* (1)
	NDM-1 and OXA-232 (2)	*K. pneumoniae* (2)
	NDM-1 and OXA-181 (3)	*K. pneumoniae* (3)
	NDM-5 and OXA-232 (1)	*K. pneumoniae* (1)
	GES-5, VIM-2 and OXA-48 (1)	*C. freundii* (1)
	IMP-1 and VIM-2 (1)	*Enterobacter* species (1)
	VIM-2 and NDM-1 (1)	*E. coli* (1)
OXA-23 (15)	OXA-23 (15)	*Acinetobacter baumannii* (15)
Non-CP CRE (10)		*K. pneumoniae* (3), *Enterobacter* species (3), *E. coli* (2), *K. oxytoca* (1), *C. freundii* (1)

**Table 2:** Results from the RESIST-5 O.K.N.V.I. assay with single carbapenemase-producing isolates stratified according to the type of carbapenemase.

	Number (%) of Clinical Isolates Tested by RESIST-5							
	KPC (*n* = 40)	NDM (*n* = 41)	VIM (*n* = 42)	IMP (*n* = 45)	OXA-48-Like (*n* = 40)	GES (*n* = 15)	OXA-23 (*n* = 15)	Non-CP CRE (*n* = 10)
KPC	40 (100)	0 (0)	0 (0)	0 (0)	0 (0)	0 (0)	0 (0)	0 (0)
NDM	0 (0)	40 (97.6)	0 (0)	0 (0)	0 (0)	0 (0)	0 (0)	0 (0)
VIM	0 (0)	0 (0)	39 (92.9)	0 (0)	0 (0)	0 (0)	0 (0)	0 (0)
IMP	0 (0)	0 (0)	0 (0)	45 (100)	0 (0)	0 (0)	0 (0)	0 (0)
OXA-48-like	0 (0)	0 (0)	0 (0)	0 (0)	40 (100)	0 (0)	0 (0)	0 (0)
Negative	0 (0)	1 (2.4)	3 (7.1)	0 (0)	0 (0)	15 (100)	15 (100)	10 (100)

**Table 3:** Sensitivity, specificity, positive predictive value and negative predictive value of RESIST-5 O.K.N.V.I. for identifying five targeted carbapenemases.

Carbapenemase Genes	Sensitivity, % (*n*/*n*)	Specificity, % (*n*/*n*)	Positive Predictive Value, % (*n*/*n*)	Negative Predictive Value, % (*n*/*n*)
*bla*_KPC_	100 (51/51)	100 (217/217)	100 (51/51)	100 (217/217)
*bla*_IMP_	100 (46/46)	100 (222/222)	100 (46/46)	100 (222/222)
*bla*_NDM_	97.6 (59/60)	100 (208/208)	100(59/59)	99.5 (208/209)
*bla*_VIM_	93.3 (42/45)	100 (223/223)	100 (42/42)	98.7 (223/226)
*bla*_OXA-48-like_	100 (47/47)	100 (221/221)	100 (47/47)	100 (221/221)
Total	98.4 (245/249)	100 (1091/1091)	100 (245/245)	99.6 (1091/1095)

## Data Availability

The datasets used/or analyzed during the current study available from the corresponding author on reasonable request.

## Supplementary Materials

The following are available online at https://www.mdpi.com/2079-6382/10/4/460/s1, Table S1: List of the isolates included in this study stratified according to the resistant genotype, Table S2: Primers used in this study.
