# Supplementary material for: Performance Evaluation of the Newly Developed In Vitro Rapid Diagnostic Test for Detecting OXA-48-Like, KPC-, NDM-, VIM- and IMP-Type Carbapenemases: The RESIST-5 O.K.N.V.I. Multiplex Lateral Flow Assay
Source: Antibiotics (Basel). 2021 Apr 19;10(4):460. doi: 10.3390/antibiotics10040460 (PMC8103512; doi:10.3390/antibiotics10040460)
Supplement: Supplementary file 1 [file antibiotics-10-00460-s001.zip › antibiotics-1188068-Supplementary Table S1 & S2.pdf]

Table S1. List of the isolates included in this study stratified according to the resistant genotype.

	Species	Origin	Disk Diffusion Test (mm)	
			Imipenem	Meropenem
GNSEV_NCP-CRE_1	*E. coli*	Wound	20	21
GNSEV_NCP-CRE_2	*E. coli*	Wound	22	21
GNSEV_NCP-CRE_3	*K. pneumoniae*	Wound	21	20
GNSEV_NCP-CRE_4	*K. pneumoniae*	Sputum	19	18
GNSEV_NCP-CRE_5	*K. pneumoniae*	Wound	16	16
GNSEV_NCP-CRE_6	*Enterobacter spp.*	Wound	16	17
GNSEV_NCP-CRE_7	*K. oxytoca*	Urine	19	23
GNSEV_NCP-CRE_8	*C. freundii*	Urine	22	21
GNSEV_NCP-CRE_9	*Enterobacter spp.*	Wound	23	21
GNSEV_NCP-CRE_10	*Enterobacter spp.*	Wound	22	21

Table S2. Primers used in this study.

Target	Primer	Nucleotide Sequence	Reference
bla$_{KPC}$	KPC-F	ATGTCACTGTATCGCCGTCT	This study
	KPC-R	TTTTCAGAGCCTTACTGCCC	
bla$_{NDM}$	NDM-F	GCCCAATATTATGCACCCGG	3
	NDM-R	CTCATCACGATCATGCTGGC	
bla$_{VIM}$	VIM-2F	ATCATGGCTATTGCGAGTCC	3
	VIM-2R	ACGACTGAGCGATTTGTGTG	
bla$_{IMP}$	IMP-1F	AAGGCGTTTATGTTCATACTTCG	3
	IMP-1R	TTTAACCGCCTGCTCTAATGTAA	
bla$_{OXA-48-like}$	OXA-48-F	AGCAAAGGAATGGCAAGAAA	This study
	OXA-48-R	TCATCAAGTTCAACCCAACC	
